# Highly fluorescent method for ultra-sensitive estimation of delafloxacin in human plasma: greenness assessment

**DOI:** 10.1186/s13065-026-01746-9

**Published:** 2026-02-25

**Authors:** Baher I. Salman

**Affiliations:** Pharmaceutical Analytical Chemistry Department, Faculty of Pharmacy, Al-Azhar University, Assiut Branch, 71524 Assiut, Egypt

**Keywords:** DEL, Human plasma, Fluorescence, AGREE, GAPI, Pharmaceutical dosage form

## Abstract

A simple, rapid, sensitive, and reliable spectrofluorimetric approach has been developed for the analysis of delafloxacin (DEL) in human plasma. The proposed method is based on the formation of a micellar fluorescent complex through the interaction of DEL with 2% sodium dodecyl sulfate (SDS) using a phosphate buffer (pH 4.2), producing a pronounced emission at 470 nm upon excitation at 380 nm. The experimental conditions that affected the one-pot synthesis were investigated and optimized. The calibration curve exhibited a linear relationship over a concentration range of 2.0–120.0 ng mL^–1^, with detection (LOD) and quantitation limits (LOQ) 0.6 and 1.9 ng mL^–1^, respectively. The designed approach was validated following the ICH and FDA guidelines, producing a precise analysis of DEL in its tablets and plasma samples with high recovery percentage. The results provide that the method possesses excellent eco-friendly properties, highlighting its strong compatibility with the principles of green chemistry.

**Supplementary Information:**

The online version contains supplementary material available at 10.1186/s13065-026-01746-9.

## Introduction

**Fig. 1:**
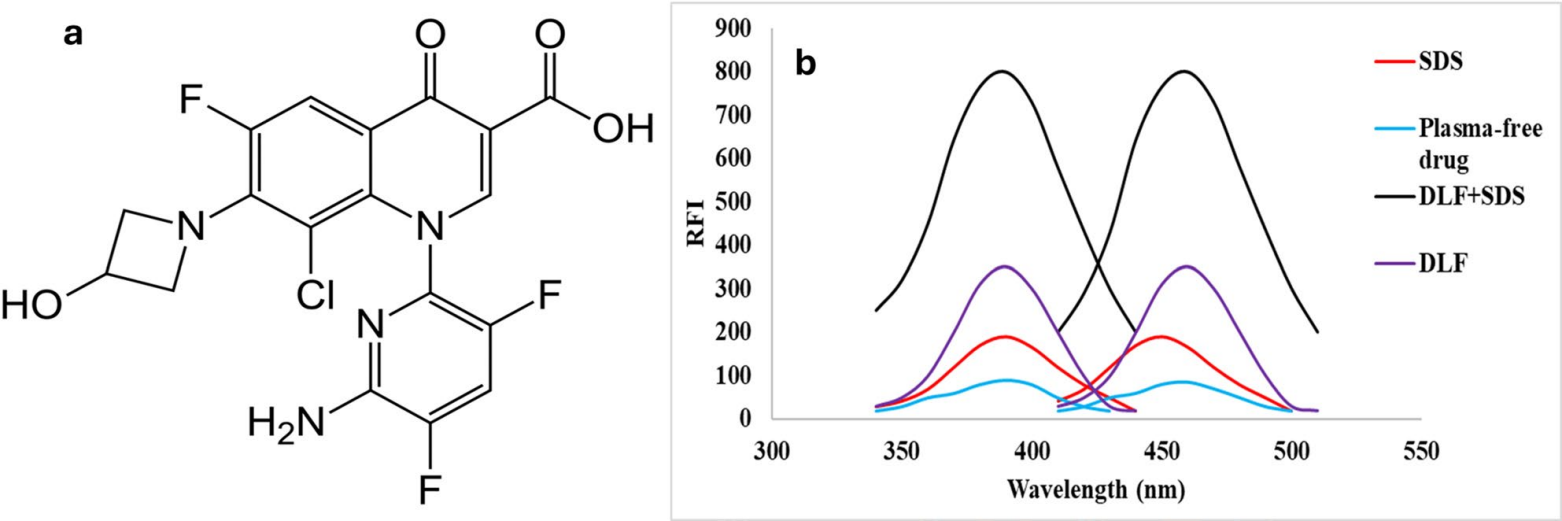
**a** Chemical structure of DEL and **b** Fluorescence spectra of reaction of DEL (90 ng mL^− 1^) with 2%SDS

The increasing prevalence of antibiotic resistance necessitates the development of new drugs with enhanced efficacy [1]. The United Nations (UN) has proclaimed that without the advancement of new antibacterial compounds, the total global mortality could surge to 10 million human deaths each year by 2050 [2]. Delafloxacin (DEL, Fig. 1a**)** has been developed and has recently gained approval from the US-FDA as a fluoroquinolone with a distinctive non-zwitterionic chemical structure. DEL can simultaneously inhibit topoisomerases II and IV, which are essential for bacterial activity [3]. This capability consequently broadens its activity spectrum against pathogens that exhibit clinical resistance. DEL has been authorized for the management of community-acquired pneumonia, a significant factor in mortality rates associated with bacterial infections on a global scale. It is also regarded as one of the most efficacious antibacterial agents for treating skin and skin structure infections [4–7]. Consequently, there is a pressing need for the development and synthesis of new molecular entities that possess improved susceptibilities and unique chemical structures [8, 9]. Even then, several approaches have been published for the estimation of DEL, including chromatographic [10–13], spectrofluorimetric [14], and potentiometric methods [15]. However, previously published methods present limitations; for example, HPLC is costly and has a low environmental profile due to high organic solvent consumption [11–13, 16, 17]. The proposed technique is based on SDS micelle–induced enhancement of the native fluorescence of DEL, which is fundamentally different from the fluorescence reaction approach using carbon quantum dots [14]. This eliminates the need for nanomaterial preparation, characterization, and stability study for quantum dots. The spectrofluorimetric technique provides a simpler, faster, more cost-effective, and highly reproducible analytical procedure [18–21].

In contrast, the proposed spectrofluorimetric method provides significantly enhanced sensitivity, allowing for more accurate quantification at lower concentration levels equal to 0.6 ng mL^− 1^. This makes the method especially useful for stability studies, low-dose formulations, and content uniformity testing. In addition, the fluorescence measurements offer greater discrimination (Higher selectivity and cheap method) against common formulation excipients compared to the reported methods [11–13, 16, 17].

The creation of innovative analytical techniques that align with the green analytical chemistry (GAC) principles can also be approached in various ways, including reducing the sample size, implementing simple and direct sample preparation [22]. Additionally, the remediation of waste materials serves as another strategy for GAC. To this end, a variety of greenness assessment tools have been developed to evaluate the environmental performance of analytical methods.

The objective of this study was to develop a green, low-cost, and sensitive fluorimetric method that is economical and environmentally sustainable for the determination of DEL in various matrices. The effectiveness of the proposed tool was tested in evaluating the uniformity of content in tablet dosage forms and in the therapeutic drug monitoring of DEL in human plasma. Furthermore, this approach employs green analytical procedure index (GAPI) and Analytical Greenness Calculator (AGREE) tools to assess its environmental greenness, efficiency, and overall sustainability.

### Experiments

#### Instruments

FS-2 spectrofluorometer was utilized and the slit width was adjusted at 5 nm. Additionally, a 1 cm quartz cell was incorporated into the system, which bears the serial number 1,304,002 (Sinco, Korea). All pH measurements were performed using a Jenway 3510 pH meter (Jenway, UK), a Grant XUBA3 ultrasonic sonicator (UK), and a laboratory centrifuge Sigma 2-16KHL (Germany).

### Materials and reagents

Delafloxacin powder (99.0% purity) was gently provided by (Melinta Therapeutics, New Jersey, USA). Baxdela^®^ tablets and vials were obtained from a pharmacy in Riyadh, Saudi Arabia. Sodium dodecyl sulphate (2%w/v SDS, ≥ 99.0% purity), tween 80 (2%v/v, 98% purity), 5%v/v polyethylene glycol 400 (99.0% purity), ethanol (≥ 99.80% purity), acetone (99.0% purity) carboxy methyl cellulose (CMC 2%w/v), methanol (≥ 99.90% purity), acetonitrile (≥ 99.9% purity), (2%w/v) cyclo-dextrin, boric acid (≥ 99.50% purity), acetic acid (≥ 99.7% purity), hydrochloric acid (37% purity), phosphoric acid (85% purity) and sodium hydroxide (≥ 98% purity) obtained from El-Nasr Co, (Cairo, Egypt). The stock solution (50 µg mL^− 1^) by dissolving five milligrams of authentic DEL in distilled water using sonication for 5 min. Further dilutions were performed using distilled water to achieve the concentration range [14].

### Application to pharmaceutical dosage forms

Ten Baxdela^®^ 450 mg tablets were weighed, finely crushed, and a powder equivalent to 10 mg of DEL was transferred into a 100 mL volumetric flask. The powder was mixed with 20 mL of methanol and sonicated for 5 min. The solution was then diluted to 100 mL with double-distilled water and filtered through a 0.45 μm membrane filter for 10 min. The resulting filtrate was subsequently subjected to the fluorimetric analytical procedure.

For (Baxdela^®^ 300 mg vials), the contents of five vials were accurately measured and combined. A portion of this mixture, equivalent to ten milligrams of DEL, was subsequently dissolved in 100 mL of distilled water.

To assess content uniformity, testing was conducted in accordance with USP [23, 24].

### Analysis of DEL in spiked human plasma

Human plasma samples were obtained from six healthy human volunteers (aged from 24 to 35 years old). The plasma analysis was conducted using the ethical committee of Al-Azhar University, Assiut branch No. ZA-AS/PH-REC/70/2024. 0.5 mL of plasma samples was combined with 0.5 mL of DEL standard solution and 2.0 mL of methanol into centrifugation test tubes. The mixture was adjusted to 10.0 mL with distilled water. Ultimately, the samples were centrifuged at 4500 rpm for 15 min. The resulting supernatant was subsequently subjected to the fluorimetric analytical procedure.

### Fluorimetric analytical procedure

Multiple aliquots of DEL were transferred into 10 mL volumetric flasks to achieve the calibration range 2.0 to 120.0 ng mL^− 1^. Subsequently, 1.0 mL of a 2% SDS solution was introduced, followed by the incorporation of 1.5 mL of phosphate buffer at pH 4.2. The flasks were completed using distilled water, which was subsequently analysed at a wavelength of 470 nm (excitation 380 nm).

## Results and discussion

To develop a novel approach for the determination of DEL, this study focused on quantifying the drug in pharmaceutical formulations and human plasma using an enhanced emission band. Various analytical methods have employed surfactants to increase the fluorescence intensity of pharmaceutical compounds, thereby improving the performance of fluorimetric techniques [25–27] **(**Fig. 1b**).** It is well established that adding a surfactant above its critical micellar concentration (CMC) to a fluorophore solution can enhance both the molar absorptivity and the fluorescence quantum yield of the fluorophore. This principle has been widely applied to optimize spectrofluorimetric methods for diverse analytes [28, 29]. As illustrated in Fig. 1b, the formation of a complex between DEL and SDS micelles produced a strong emission at 470 nm (excitation at 380 nm), with a linear calibration range of 2.0–120.0 ng mL⁻¹. The proposed method demonstrates high sensitivity, a low detection limit, and precision comparable to conventional techniques, establishing it as a robust and effective approach for DEL quantification in biological fluids.

### Optimization of the proposed method

Various elements can affect the development of the relative fluorescence intensity (RFI) of the reaction such as pH, type and amount of buffer, and quantity of surfactant.

### Effect of pH and volume of buffer solution

**Fig. 2:**
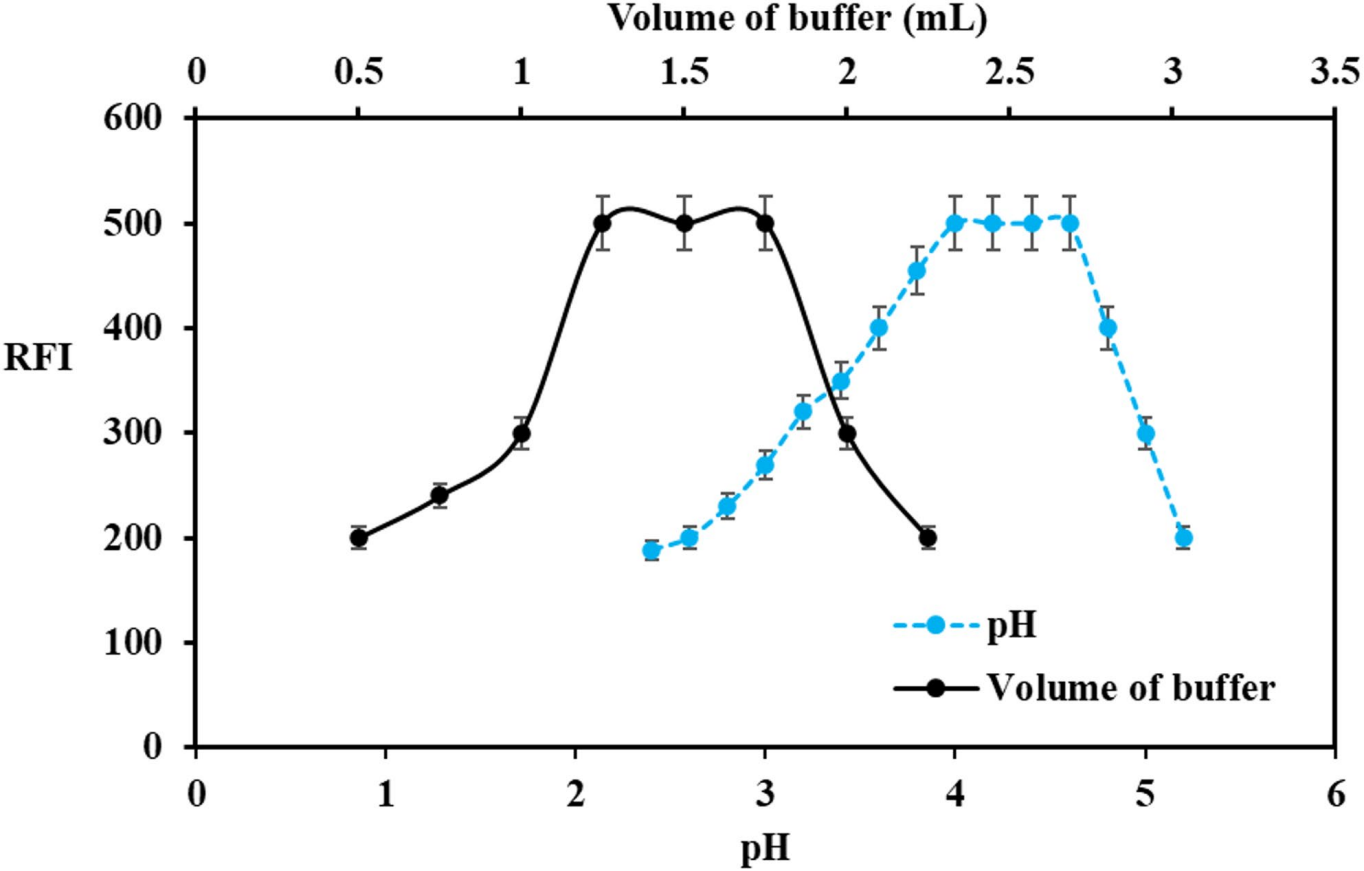
Effect of **a** pH and **b** Volume of buffer on RFI for analysis of DEL (50 ng mL^− 1^) using 2% SDS

Phosphate buffer in the range 2.5 to 5.5 was utilized for pH study (Fig. 2**)**. The effect of pH on micelle-enhanced fluorescence exhibits a peak within the pH range of 4.0 to 4.6, as illustrated in **(**Fig. 2**).** At pH 4 to 4.6, DEL predominantly exists in its protonated form due to protonation of the basic functional groups. Under these conditions, SDS (anionic surfactant) interacts electrostatically with the positively charged delafloxacin molecules, leading to the formation of a stable ion-pair/association complex. This interaction resulted in a significant enhancement in the fluorescence intensity. Therefore, pH 4.2 was selected as the optimum condition to ensure maximum interaction between DEL and SDS. Additionally, the volume of phosphate buffer at pH 4.2 produced the highest fluorescence with 1.5 ± 0.25 mL.

### Organized media

**Fig. 3:**
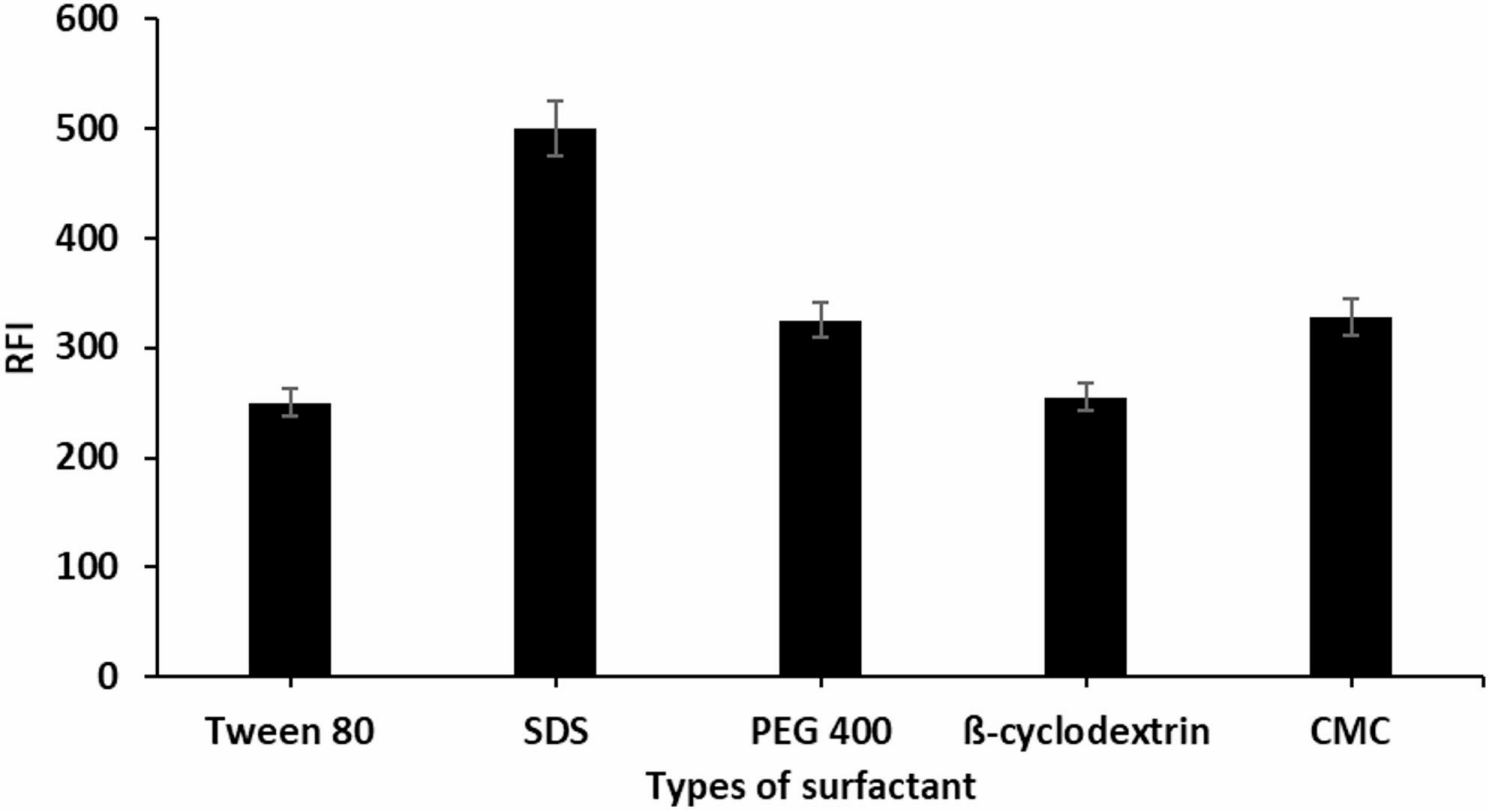
Effect of various types of surfactants with DEL (50 ng mL^− 1^) using the proposed method

The fluorescence characteristics of DEL in different micellar environments were examined using SDS, Tween-80, methyl cellulose, PEG 400 and β-cyclodextrin. All structured media resulted in a negligible increase in the native fluorescence of DEL or had no considerable impact on its RFI, while the SDS system produced a significant enhancement effect on the RFI of DEL (Fig. 3). This enhancement can be linked to DEL’s capacity to associate and bind with SDS. Within the micellar phase, SDS can create an ion-paired species with DEL through the interaction between the negatively charged sulfonyl group of SDS and the protonated tertiary amine group of DEL. The binding of micelles improved the fluorescence intensity of DEL by minimizing the electrostatic attractions and collisions among DEL molecules.

The effect of SDS volume on the RFI was examined by utilizing larger volumes of 2.0% w/v SDS. It was observed that as the volume of the SDS solution increased, there was a proportional rise in RFI up to 0.75 mL, beyond which no additional increase in RFI was observed. Consequently, 1.0 ± 0.25 mL of SDS was selected as the ideal volume for DEL determination (Fig. S1).

### Diluting solvents

**Fig. 4:**
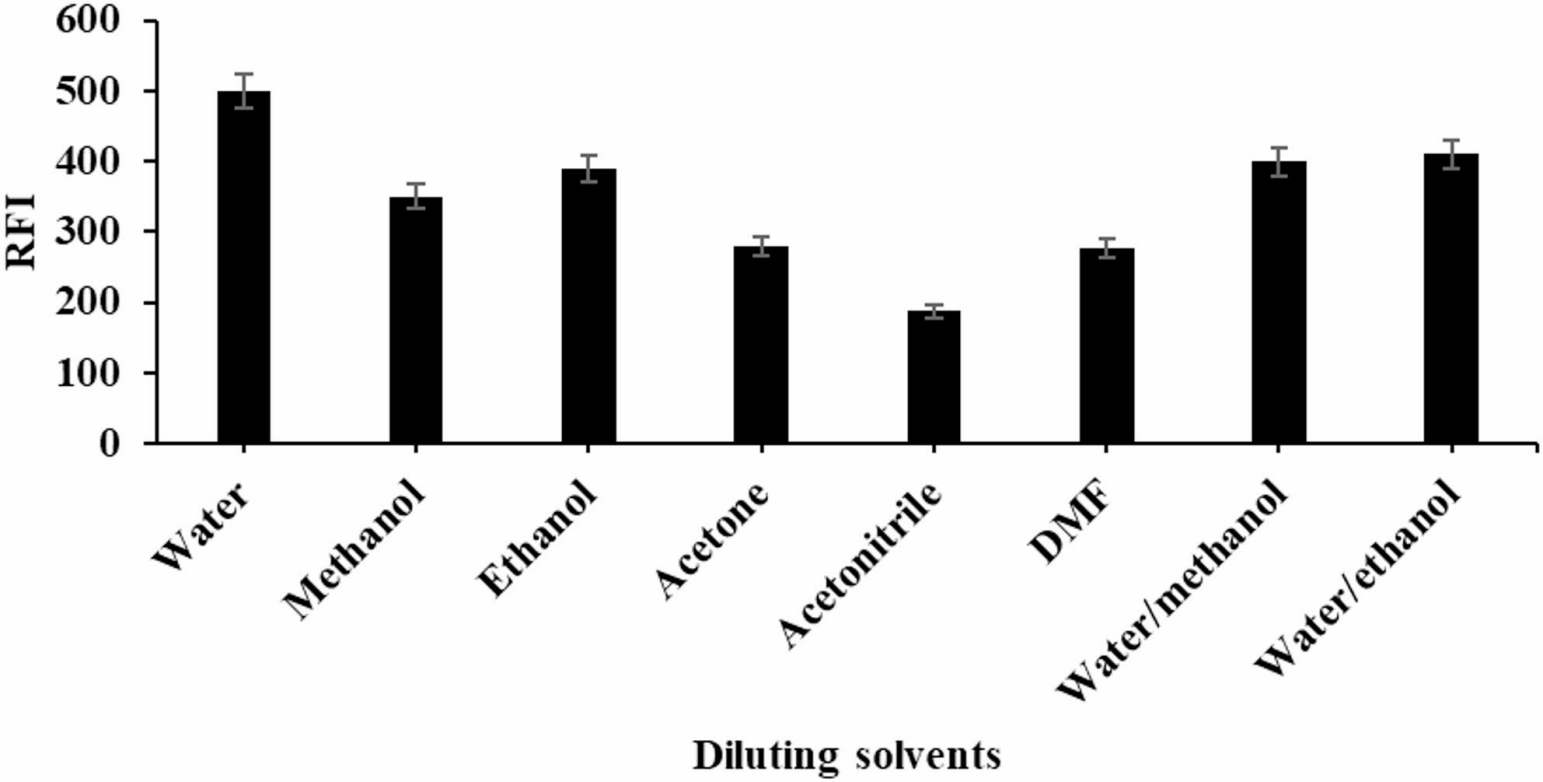
Effect of diluting solvents on RFI for analysis of DEL (50 ng mL^− 1^) using 2% SDS

The impact of various diluting solvents on the RFI of DEL was examined using distilled water, methanol, ethanol, acetonitrile, acetone, dimethyl formamide, a water-methanol mixture (80:20 v/v), and a water-ethanol mixture (80:20 v/v). It was determined that water served as the most effective solvent for dilution, yielding the highest RFI and the lowest blank reading. The short-chain alcohols (methanol and ethanol) primarily dissolve in the aqueous phase and influence the micellization process by altering the properties of the solvent. The addition of these alcohols lead to a decrease in micelle size, although this occurs alongside a gradual disintegration of the surfactant aggregate at very high concentrations [28]. Figure 4.

### Analysis and validation of the fluorimetric approach

**Fig. 5:**
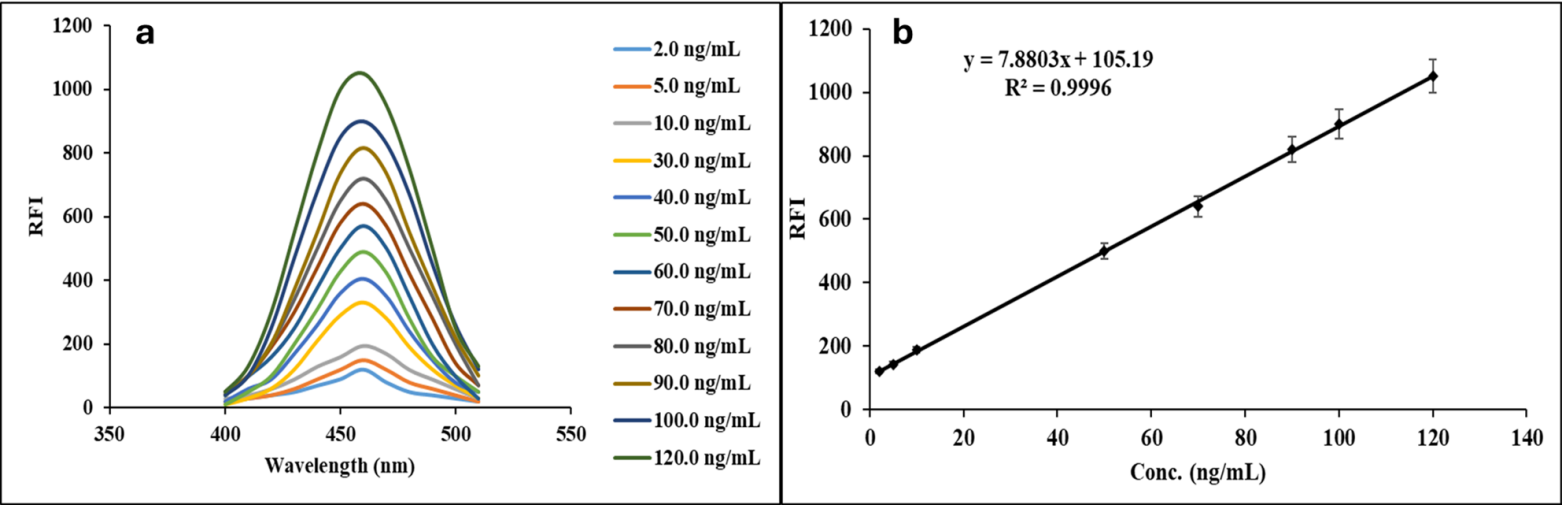
**a** Reaction of SDS with different concentration **b** calibration graph for quantification of DEL using the proposed method

The proposed fluorometric approach was validated following ICH and FDA guidelines [30, 31]. A calibration curve was generated by plotting the relative fluorescence intensity (RFI) against the concentrations of DEL (Fig. 5a).

### Linearity and range

The limit of detection and quantification (LOD and LOQ) were calculated using the slope and standard deviation (ϭ) of the curve. LOQ was computed as (10 ϭ/ slope), while LOD was calculated as (3.3 ϭ/ slope). The plot of calibration was linear over the range of 2.0–120.0 ng mL^− 1^, as shown in Fig. 5b. The calibration graph’s linearity was observed by the statistical analysis, as demonstrated by the high correlation coefficient (*r* = 0.9996). Table 1.

**Table 1:** Analytical performance results for analysis of DEL using fluorimetric approach

Parameter	Results
λ_ex_ (nm)	380
λ_em_(nm)	470
Concentration range (ng mL^− 1^)	2.0–120.0
Determination coefficient (r^2^)	0.9996
Slope	7.9 ± 0.2
Intercept	105.2 ± 2.6
SD the intercept (Sa)	1.5
LOD (ng mL^− 1^)	0.6
LOQ (ng mL^− 1^)	1.9

LOD: lower limit of detection; LOQ: lower limit of quantitation

### Accuracy and precision

The designed fluorimetric method’s accuracy was assessed at different levels (5.0, 10.0, 40.0, 80.0, and 100.0 ng mL^− 1^). The obtained results revealed high recovery percentages with low SD (Table 2), which indicates the high accuracy of the designed method. Additionally, both intraday and inter-day precision were determined to further evaluate its accuracy. The precision was examined using three replicates of three concentrations of DEL (10.0, 30.0, and 80.0 ng mL^− 1^). Table 2.

**Table 2:** Results of accuracy and precision study using the designed approach

Sample number	Taken Conc. (ng mL^− 1^)	Found Conc. (ng mL^− 1^)	% Recovery ^*^ ± RSD
1	5.0	5.1	102.0 ± 0.3
2	10.0	10.1	101.0 ± 0.2
3	40.0	40.2	100.5 ± 0.4
4	80.0	79.8	99.7 ± 0.3
5	100.0	101.6	101.6 ± 0.7
Intra-day precision	10.0	10.2	102.0 ± 0.7
	30.0	30.3	101.0 ± 0.9
	80.0	80.5	100.6 ± 0.2
Inter-day precision	10.0	10.1	101.0 ± 1.0
	30.0	30.1	100.3 ± 1.2
	80.0	79.9	99.8 ± 0.8

^*^: Average of three determinations. **RSD**: Relative standard deviation

### Stability study and matrix effect of DEL in human plasma

The stability of DEL in human plasma was studied as follow, three concentrations were used to investigate the stability of DEL in human plasma, including low-quality control (LQC 5.0 ng mL^− 1^), medium-quality control (MQC 60.0 ng mL^− 1^), and high-quality control (HQC 100.0 ng mL^− 1^) levels. These concentrations were employed to measure stability for three freeze-thaw cycles (-24 °C), long-term stability (1 month at -24 °C), short-term stability (12 h at -24 °C), and post-preparative. The obtained recovery percentages ranged from 96.6 ± 1.5 to 98.8 ± 1.8% which indicates to the high stability of DEL in human plasma. Table S1 provides all the findings from the analysis of the stability effect on human plasma.

In addition, the impact of the plasma matrix was also examined concerning DEL determination (2.0, 50.0, and 100.0 ng mL^− 1^). The mean recovery obtained from six different plasma lots ranged from 97.0% to 98.5%, with RSDs ranging from 1.3% to 1.9%, which are well below 15%, indicating no significant matrix effect. Table S2.

### Robustness study

The robustness of the novel method was evaluated through minor adjustments in pH, buffer volume, and the volume of 2% SDS as outlined in the methodology. The findings presented in Table S3 indicate that each alteration in the analytical conditions leads to negligible variations in the RFI. No substantial changes in the results were detected following slight variations as indicated in Table S3.

### Selectivity study

The selectivity of the introduced green approach was evaluated in comparison to various excipients and different fluoroquinolones. As indicated in (Table S4), no interferences were detected in the proposed method.

### Applications of the inventive approach

The suggested methodology was employed for quantifying DEL in its commercial formulations. The percentage of recoveries ± RSD obtained was found to be 102.1 ± 1.0 for Baxdela^®^ tablets and 102.6 ± 0.9 for vials, respectively compared to reported method [14]. The comparison was performed using the same pharmaceutical formulation, and statistical evaluation (Student’s *t*-test and F-test) showed no significant difference between the two methods at the 95% confidence level. These findings confirm that the proposed method provides accuracy and precision comparable to those of an established separation-based method. Table 3.

**Table 3:** Comparison between the proposed spectrofluorimetric and reported method for determination of DEL in their pharmaceutical dosage forms

Dosage form	%Recovery*± SD		t-value	F-value
	Proposed	Reported [14]		
Baxdela^®^ 450 mg tablets	102.1 ± 1.0	101.9 ± 0.7	0.96	1.75
Baxdela^®^ 300 mg vial	102.6 ± 0.9	102.4 ± 1.4	1.20	2.13

The tabulated t- and F- values at 95% confidence limit are 2.78 and 6.39, respectively. * Mean of five determinations

Maintaining uniformity of dosage units is a key aspect of quality control in pharmaceutical formulations, ensuring that each tablet in a batch contains the specified amount of active pharmaceutical ingredient as indicated on the product label. This consistency is vital for achieving the desired therapeutic effect and for preventing potential risks associated with under- or overdosing, which could endanger patient safety. In this work, content uniformity was studied using standardized procedures in strict compliance with USP guidelines [23, 24]. Table 4.

**Table 4:** Results of consistency test for quantifying DEL using the proposed method

Tablet No.	% Labeled claim
	Baxdela^®^ 450 mg tablets
1	101.6
2	101.4
3	101.7
4	100.2
5	101.6
6	100.9
7	102.9
8	102.4
9	101.2
10	100.1
Mean	101.4
SD	0.8
RSD	0.8
Acceptance value (AV)*	2.0
Max. allowed AV (L1)*	15

* Acceptance value = 2.4 × SD

Furthermore, the green proposed method was successfully applied to the quantification of DEL in spiked human plasma. The findings (Table 5) indicate elevated recovery percentages of DEL achieved through the proposed method from human plasma.

**Table 5:** Application of the proposed method for analysis of DEL in plasma samples

Added conc. (ng mL^− 1^)	Found Conc. (ng mL^− 1^)	% Recovery ^*^± RSD
5.0	4.9	98.0 ± 0.9
10.0	9.7	97.0 ± 1.5
20.0	19.6	97.5 ± 1.7
50.0	49.2	98.4 ± 1.4
80.0	78.7	98.3 ± 1.2
100.0	98.5	98.5 ± 1.8

^*^: Average of three determinations

### Assessment of the greenness profile

Two modern tools (GAPI and AGREE) were developed for the assessment of the greenness profile of the proposed method. Green chemistry is a fundamental aspect of sustainable chemistry, concentrating on making chemical reactions and products more environmentally benign, considering factors such as molecular efficiency, energy efficiency, safe reactants, renewable resources, and pollution prevention. The evaluation of the green method indicated favourable results, demonstrating low environmental affect using different tools. Table 6.

**Table 6:**
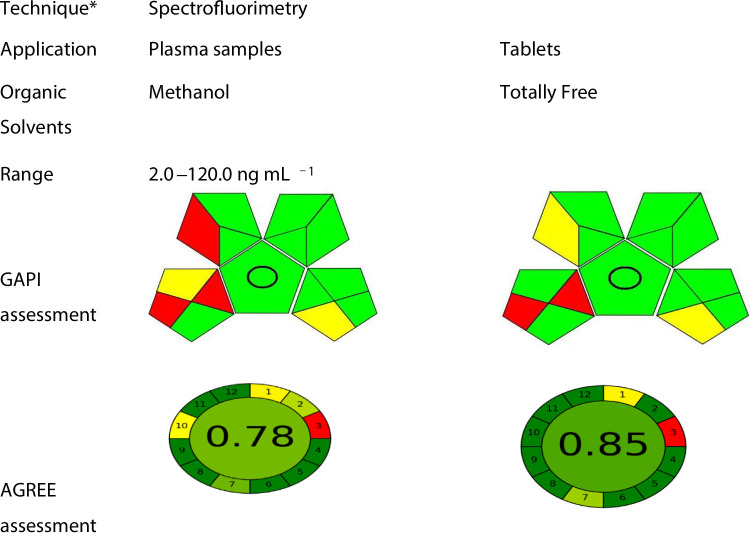
The results of the greenness study for assessments of the proposed method

### Assessing the proposed method for DEL Estimation relative to some reported methods

The performance metrics of the fluorimetric method were evaluated against other reported techniques, with the findings compiled in Table S5. This table demonstrates that the proposed method shows greater sensitivity than other methods with LOQ equal to 1.9 ng mL^− 1^. Furthermore, the recently developed method presets multiple benefits over previously established techniques. These advantages encompass the utilization of low cost and eco-friendly solvents. Table S5.

## Conclusion

An efficient, established, and cost-effective green micellar complexation method has been developed and validated for the quantification of DEL in different matrices. This technique was successfully utilized in dosage forms and human plasma. Furthermore, the environmental sustainability of this study aligns with the principles of green analytical chemistry.

## Supplementary Information

Below is the link to the electronic supplementary material.

Supplementary Material 1

## Data Availability

The datasets used and/or analyzed during the current study are available from the corresponding author upon reasonable request.
