# Supplementary material for: Highly fluorescent method for ultra-sensitive estimation of delafloxacin in human plasma: greenness assessment
Source: BMC Chem. 2026 Feb 25;20(1):47. doi: 10.1186/s13065-026-01746-9 (PMC12955026; doi:10.1186/s13065-026-01746-9)
Supplement: Supplementary file 1 — Supplementary Material 1 [file 13065_2026_1746_MOESM1_ESM.docx]

**Highly Fluorescent Method for Ultra-sensitive Estimation of
Delafloxacin in Human Plasma: Greenness Assessment**

**Baher I. Salman^1^***

*^1^ Pharmaceutical Analytical Chemistry Department, Faculty of Pharmacy, Al-Azhar University, Assiut Branch, Assiut, 71524, Egypt**.*

***Corresponding author: Baher I. Salman (bahersalman@azhar.edu.eg)**


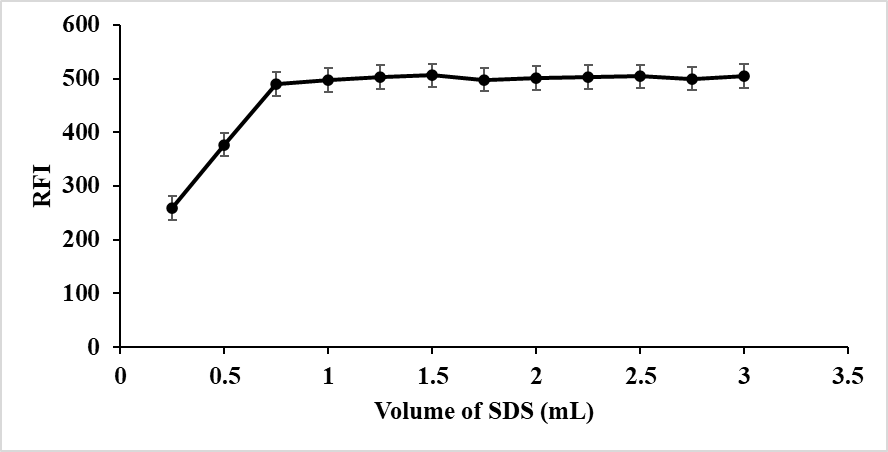


**Figure S1:** Effect of volume of SDS on the RFI of DEL (50 ng mL^-1^)

**Table S1:** Results of stability study of DEL in human plasma.

|  | **LQC**  **5.0 ng mL^-1^** | **MQC**  **60.0 ng mL^-1^** | **HQC**  **100.0 ng mL^-1^** |
| --- | --- | --- | --- |
| Three Freeze–thaw cycle stability (-24°C) | 96.9 ± 1.7 | 97.3 ± 1.5 | 97.12 ± 1.9 |
| Long-term stability (1 month at -24°C) | 98.8 ± 1.8 | 97.5 ± 1.1 | 97.91 ± 1.0 |
| Short-term stability (12 h at -24°C) | 98.41 ± 1.6 | 98.6 ± 1.8 | 98.15 ± 1.4 |
| Post-preparative stability (6 h at room temperature 25 °C) | 97.7 ± 1.5 | 98.4 ± 1.2 | 97.88 ± 1.1 |
| Post-preparative stability (12 h at room temperature 25 °C) | 97.3 ± 1.6 | 96.6 ± 1.5 | 98.47 ± 1.0 |

Data presented as recovery (%) ±SD (n = 5).

**Table S2:** Matrix effect and bioanalytical validation of the fluorimetric method for analysis DEL in human plasma.

| **Inter-day assay (n=18)** | | **Intra-day assay (n=6)** | |  | |  |
| --- | --- | --- | --- | --- | --- | --- |
| **Precision**  **(CV %)** | **Accuracy (%)** | **Precision**  **(CV %)** | **Accuracy**  **(%)** | | **Conc.**  **(ng mL^-1^)** | |
| 1.9 | 97.0 | 1.4 | 97.7 | | 2.0 | |
| 1.4 | 97.9 | 1.7 | 98.2 | | 50.0 | |
| 1.3 | 98.5 | 1.4 | 98.1 | | 100.0 | |

**Table S3:** Robustness of the proposed approach for analysis of DEL (50.0 ng mL^-1^).

|  | |  | **Variations** |
| --- | --- | --- | --- |
| **% Recovery ^a^ ± RSD** | |  |  |
| 101.4 ± 0.1 | |  | **Optimum condition** |
| **1- Effect of Ph** | | | |
| 100. 4 ± 0.3 | | 4.0 | |
| 100.0 ± 0.7 | | 4.4 | |
| **2- Volume of buffer (mL)** | | | |
| 100.1 ± 0.5 | | 1.25 | |
| 100.2 ± 0.7 | | 1.75 | |
| **3- Volume of 2 % SDS** | | | |
| 100.2 ± 0.3 | | 0.75 | |
| 99.9 ± 0.2 | | 1.25 | |

**^a^:** Mean of three determinations.

**Table S4:** Selectivity study of the proposed method

|  | **Recovery* ± RSD** |
| --- | --- |
| **Mannitol (10 mg)** | 102.1 ± 0.2 |
| **Talc (10 mg)** | 101.7 ± 0.6 |
| **Starch (100 mg)** | 100.2 ± 0.2 |
| **Sucrose (10 mg)** | 100.6 ± 0.3 |
| **Magnesium stearate (10 mg)** | 101.3 ± 0.4 |
| **Sodium chloride (10 mg)** | 102.2 ± 0.5 |
| **Levofloxacin (100 ng mL^-1^)** | 101.8 ± 0.9 |
| **Ciprofloxacin (100 ng mL^-1^)** | 100.5 ± 1.4 |
| **Moxifloxacin (100 ng mL^-1^)** | 101.4 ± 1.1 |
| **Gatifloxacin (100 ng mL^-1^)** | 100.1 ± 0.8 |

*: Mean of three determinations

**Table S5:** Comparison study between the proposed method and the reported methods

| **Method** | **Linear range** | **LOQ** | **Applications** | **Ref.** |
| --- | --- | --- | --- | --- |
| **The proposed method** | **2.0 – 120.0 (ng/ml)** | **1.9** | **Commercial tablets, uniformity test and human plasma** |  |
| Chromatography | 25.0 – 1000.0 (ng/band) | 25.0 | Pharmaceutical formulation | [10] |
| Chromatography | 3.5 – 5000.0 (ng/ml) | 3.5 | Plasma samples | [11] |
| Chromatography | 2.9 – 6666.0 (ng/ml) | 2.9 | Rat plasma | [12] |
| Chromatography | 100.0 – 2500 (ng/ml) | 100.0 | Human plasma | [13] |
| Spectrofluorimetry | 0.5 – 100.0 (ng/ml) | 0.27 | Dosage form and human plasma | [14] |
